# 3D CNT/MXene microspheres for combined photothermal/photodynamic/chemo for cancer treatment

**DOI:** 10.3389/fbioe.2022.996177

**Published:** 2022-09-19

**Authors:** Wei Gao, Weihao Zhang, Haipeng Yu, Wenge Xing, Xueling Yang, Yongguang Zhang, Chunyong Liang

**Affiliations:** ^1^ Department of Interventional Therapy, Tianjin Medical University Cancer Institute and Hospital, National Clinical Research Center for Cancer, Tianjin, China; ^2^ Key Laboratory of Cancer Prevention and Therapy, Tianjin, China; ^3^ Tianjin’s Clinical Research Center for Cancer, Tianjin, China; ^4^ State Key Laboratory of Metastable Materials Science and Technology, Yanshan University, Qinhuangdao, China; ^5^ School of Materials Science and Engineering, Hebei University of Technology, Tianjin, China

**Keywords:** photodynamic therapy, photothermal therapy, 3D CNT/MXene, synergistic therapy, chemotherapy, doxorubicin

## Abstract

MXene nanosheets have shown exciting potential in nanomedicine because of their large surface area, intense near-infrared (NIR) absorbance, and good biocompatibility. However, their development in the direction of treating tumors is constrained by the limitations of existing design methodologies. These methodologies lack control over the size and distribution of tumors. Moreover, their photodynamic therapy (PDT) effect is poor. To address this unmet medical need, a simple strategy that processes MXene with carbon nanotube (CNT) into a three-dimensional (3D) honeycomb structure having anti aggregation capacity was established. The structure can be used in disease phototherapy against tumors, bacteria, and viruses, such as photothermal therapy (PTT), photodynamic therapy (PDT), and multimodal synergistic therapy. In the present study, 3D CNT/MXene microspheres were obtained by the template method and spray-drying method. The microspheres possessed special photothermal effects and photothermal stability under NIR laser irradiation. Furthermore, the developed microspheres could achieve a maximum of 85.6% drug loading capability of doxorubicin (DOX). Under light irradiation at 650 and 808 nm, 3D CNT/MXene microspheres could efficiently produce singlet oxygen due to the effectiveness of CNTs as carries for Titanium Dioxide (TiO_2_) photosensitizers present on the MXene surface. Furthermore, *in vitro* studies had showed that 3D CNT/MXene-DOX effectively inhibited the proliferation of HeLa cells. Hence, this study provides a promising platform for future clinical applications to realize PTT/PDT/chemotherapy combination cancer treatment based on MXene.

## Introduction

Cancer, a leading threat to human health, has become one of the most severe diseases (Henley et al., 2020). In the last decades, cancer treatment has made tremendous progress in all aspects of clinical practice. The leading cancer treatments include surgery, chemotherapy, radiotherapy, hormone therapy, and targeted therapy (Vaddepally et al., 2020). However, traditional cancer treatments have many disadvantages, including resistance to malignant tumors, drug side effects, and related toxicity (Zhang et al., 2013; Chen et al., 2017; Wang et al., 2018; Chen et al., 2019; Garcia-Cortadella et al., 2021). Photodynamic therapy (PDT) and photothermal therapy (PTT) possess several advantages like insignificant invasiveness, low toxicity, high therapeutic efficacy, limited side effects, effective selectivity, and reproducible properties. They hence have received much attention in recent years (Dolmans et al., 2003; Taratula et al., 2014; Liu et al., 2018).

Photothermal therapy, a noninvasive and localized therapeutic modality, can convert near-infrared (NIR) laser energy into hyperthermia, raising the local temperature to selective thermal ablation of cancer cells (Song et al., 2016; Zou et al., 2016; Hu et al., 2018). Photodynamic therapy is a relatively new approach in treating cancer (Ming et al., 2019; Wan et al., 2019; Zhang et al., 2019). In this approach, a photosensitizer (PS) is administered and subsequently activated by illumination with light of specific wavelengths at the target site. The activated photosensitizer induces tumor cell death and vascular shutdown by the generation of reactive oxygen species (ROS). Based on this approach, the drug delivery systems (DDS) with excellent bioavailability and superior stability upon combining with PTT and PDT can maximize the therapeutic efficacy and reduce damage to surrounding normal tissues (Liu et al., 2017). Currently, inorganic hollow or porous micro or nanostructures have sparked significant concern in relevant advanced technology fields due to their high specific surface area, thermal stability, low toxicity, and low effective density (Sun et al., 2014; Yue et al., 2015).

In recent years, layered two-dimensional (2D) nanomaterials have received tremendous research attention thanks to their unique physicochemical properties. These 2D nanomaterials, such as graphene (Choi et al., 2018; Pandey et al., 2021), black phosphorus nanosheets (Choi et al., 2018; Pandey et al., 2021), and MoS_2_ (Liu et al., 2015), promote the malignant biological behaviors of cancer cells. MXene, a new type of two-dimensional transition-metal, carbide or nitride materials (Wu et al., 2020) was discovered in 2011 (Naguib et al., 2011). MXenes are formed by selectively etching the parent MAX phase to remove the A-group element (typically Al or Ga). The 2D MXenes represent a promising modality in biomedical research and clinical applications because of their high surface area, capacity for facile functionalization, high NIR photothermal conversion performance, and superior photothermal conversion efficiency for the PTT of tumor cells (typically Al or Ga). (Lin et al., 2017).

However, recent research direction has only concentrated on enhancing the potential of MXene as a drug delivery platform for combined chemo- and photothermal therapy, the reproducibility and size distribution of MXene have been neglected in tumor therapy (Soleymaniha et al., 2019). Moreover, Ti_3_C_2_ nanosheets are predisposed to agglomerate because of their high surface energy and intrinsic van der Waals (vdW) forces preventing their uniform distribution. In order to address the above problem, Ti_3_C_2_ nanosheets are processed into a three-dimensional (3D) spheroid with a rough surface that could effectively reduce the area of contact between the MXene (Xiu et al., 2018; Fang et al., 2020). Besides, 3D architectures with a rough surface and increased surface area are effective for designing efficient platforms for sustained drug release. In this connection, it is necessary to develop novel nanostructures for MXene, such as nanotubes and spherical structures, for their utilization in cancer treatment. TiO_2_ readily forms on the surface of 3D MXene in the manufacturing process, and has great potential to serve as an effective photosensitizer for PTT-based cancer treatment (Ziental et al., 2020). Nevertheless, the limitations of TiO_2_ in photocatalytic applications are its high band gap (3.0 eV for rutile and 3.2 eV for anatase) requiring high-energy ultra-violet (UV) radiations for its activation, and fast recombination of photogenerated electrons and holes (Cheng et al., 2009; Khataee et al., 2013; Peng et al., 2015).

The semiconductor material TiO_2_ has been considered as a new type of photosensitizer (PS), as they are able to catalyze H_2_O_2_ into O_2_. It is reported that H_2_O_2_ is abundant in cancer microenvironment and is an appropriate source for the production of O_2_ within tumors (Zheng et al., 2019). Upon the absorption of a photon with energy that is equal to or higher than this value, TiO_2_ can be excited to produce negative electrons in the conduction band, leaving positive holes in the valence band. These charge carriers react with surrounding water or oxygen to yield cytotoxic ROS such as hydroxyl (-OH), hydrogen peroxide (H_2_O_2_), and superoxide (-O2-). Both the generated holes and ROS are strong oxidizers that can attack cell membranes and other cellular components, leading to apoptosis of cancer cells (Feng et al., 2015). Carbon nanotube (CNT), as a sensitizer, has been shown to expand the TiO_2_ absorption range to the visible light region (Wang et al., 2005; Dai et al., 2009). Moreover, it is an excellent electronic conductor that can contribute to the transfer of the electrons suppressing the recombination of photogenerated electron-hole pairs. In addition, CNTs have high optical absorbance in the NIR region. Therefore, they dissipate a high amount of heat energy, thus inhibiting tumor cell growth at lower doses and laser intensity (Burke et al., 2009; Murakami et al., 2012). Currently, researchers working on the development of MXene for tumor treatment are focused on enhancing its chemo- and photothermal therapeutic effects while simultaneously controlling the side effects (Gazzi et al., 2019; Fang et al., 2020; Mashtalir et al., 2014; Ou et al., 2021). However, the utilization of Myxine in photodynamic therapy has been rarely reported for tumor treatment. Thus, clinical application of Memes is restricted due to the suboptimal efficacy of its current photodynamic therapy, and therefore a further in-depth study is required.

**SCHEME 1:**
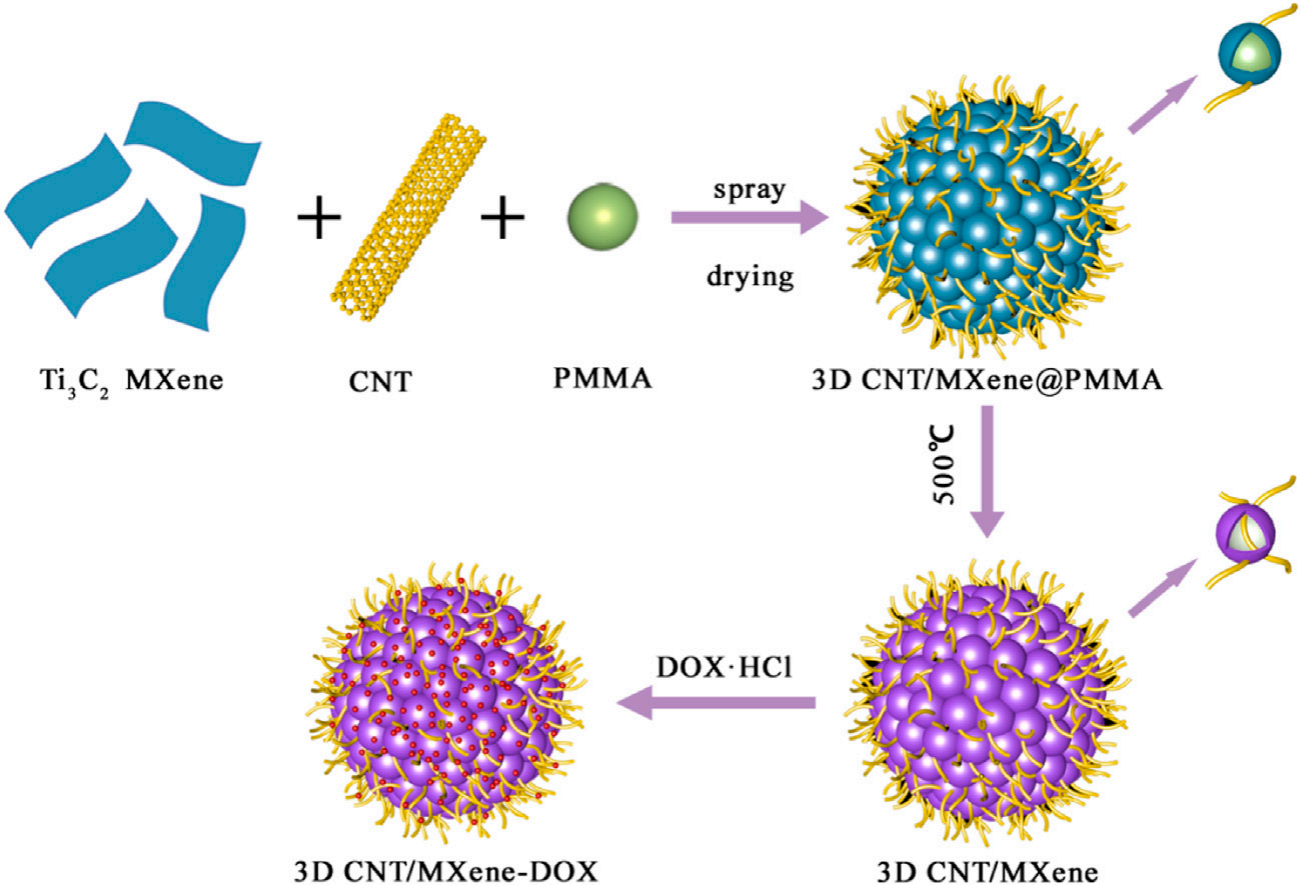
As schematic representation of fabrication process for drug-loaded 3D CNT/MXene-DOX composite microsphere.

Herein, a simple strategy that processes Ti_3_C_2_ nanosheets with CNT into a 3D honeycomb structure with anti-aggregation properties was reported, and this structure can be potentially applied in the synergistic therapy of PTT, PDT, and chemotherapy. 3D CNT/meme coated polymethyl methacrylate (PMMA) microspheres were obtained by spray drying method using PMMA as the template. During this process, titanium dioxide (TiO_2_) formed on the surface of MXene. PMMA is removed by calcination to obtain 3D CNT/MXene microspheres (Scheme 1). The obtained microspheres possessed special photothermal effects and photothermal stability under NIR laser irradiation. The developed microspheres could achieve a maximum of 85.6% drug loading capability for doxorubicin (DOX). Under light irradiation at 650 and 808 nm, 3D CNT/MXene microspheres could efficiently produce singlet oxygen because CNT improved conditions for TiO_2_ present on the MXene surface by working as carriers for TiO_2_ photosensitizers. 3D CNT/MXene microspheres have larger surface area, providing excellent sites of drug attachment for anti-cancer drugs. This study combines the advantages of NIR light-triggered photothermal nano agents and photosensitizers. Moreover, the strategy utilized here provides a promising platform to realize PTT/PDT/chemotherapy combination cancer treatment based on Ti_3_C_2_ nanosheets.

## Methods

### Synthesis of 3D CNT/MXene microspheres

200 mL mixed aqueous solution of MXene nanosheets (Beijing Beike New Material Technology Co. Ltd.) and CNT (Nanjing XFNANO Materials Tech Co. Ltd.) was first prepared in a volume ratio of 1:2. The resulting solution was vigorously stirred for 30 min for the uniform distribution of components. To this, 100 mL PMMA dispersion (12 mg mL^−1^ concentration) was added dropwise and further stirred for 10 min. The obtained solution was fed into the spray-dryer (Beijing Holves Biotechnology Co. Ltd.) at 120°C, followed by combustion at 500°C for 3 h under Ar to obtain 3D CNT/MXene microspheres.

### Characterization

Scanning electron microscopy (SEM) and transmission electron microscopy (TEM) images were taken by Hitachi S-4800 and JEOL JEM2100F, respectively. X-ray diffraction analysis of the sample was carried out using an X-ray diffractometer meter (XRD, D8 Focus, Cu Ka radiation, Bruker, Germany). Brunauer-Emmett-Teller (BET, V-Sorb 2800P) technique was adopted to obtain the specific surface area (SSA). The UV-Visible absorption spectral measurements were carried out with a ucleic-acid/protein analyzer (Beckman DU-800, CA, United States). The LSM 800 (Beijing Precise Instrument Company, China) was used to observe and record the confocal laser scanning microscope (CLSM) images.

### Preparation of 3D CNT/mxene-DOX

4.0 mg 3D CNT/MXene was dispersed in a 10 mL phosphate-buffered saline (PBS) solution of DOX (DOX concentration = 0.5 mg mL^−1^, pH = 7.4). The resulting mixture was then stirred for 24 h on a magnetic stirrer at 37°C. After that, the mixture was centrifuged for 10 min at 6,000 rpm. The samples were washed with PBS and dried for 24 h at 80°C. All the supernatant was collected to evaluate the DOX loading capacity by using a UV-Vis spectrophotometer. DOX drug-loading capacity was measured using the following equation:

Drug−loading capacity=(total DOX−unbound DOX)÷total 3D CNT/MXenes

### Drug release

3D CNT/MXene-DOX was added into two different 10 mL PBS solutions of pH 7.4 and 5. The obtained two suspensions were incubated in a shaking bath (150 rpm) for 3 h at 37°C, and 3 mL supernatant was collected from each. In the meantime, fresh PBS was added to keep the volume constant (3 mL). The amount of DOX released was measured using UV-Vis spectroscopy at different time intervals.

### Photothermal effect

1.0 mL 3D CNT/MXene aqueous dispersions (0.125, 0.25, and 0.5 mg mL^−1^) were irradiated with 808 nm NIR laser (at different power densities of 0.5, 1.0, and 1.5 W cm^−2^) for 5 min. The temperature change was also monitored in real-time with an infrared imaging system. The photo stability of 3D CNT/MXene was tested out under 808 nm laser irradiation for five ON/OFF cycle irradiation. The calculation formula of photothermal conversion efficiency (*η*) is:

$$\eta =\frac{hs\left(T\max-Tsurr\right)-Q\;Dis}{I\left(1-10^{-A808}\right)}$$ (1)

Where *h* indexes the heat transfer coefficient; *s* represents the surface area of the used holder; T_max_ and T_surr_ denote the maximum steady-state temperature and room temperature of the ambient environment, respectively; Q_Dis_ is the heat wastage form the light loss of the solvent and holder; *I* index laser intensity, A_808_ represents the 808 nm absorbance of 3D CNT/MXene.

### Cell culture

HeLa cells were obtained from the Tianjin Cancer Hospital. Cells were cultured in Dulbecco’s Modified Eagle Medium (DMEM, Solarbio Science and Technology Co. Ltd.) at 37°C in a humidified incubator containing 5% CO_2_ for 24 h, followed by treatment. The HeLa cells were incubated with fluorescein is thiocyanate (FITC)-labeled 3D CNT/MXene (500 μL, 1.0 mg mL^−1^). HeLa cells were harvested after 4, 8, and 12 h by washing them 3 times with PBS. Afterwards, HeLa cells were fixed with 4% paraformaldehyde, and nuclei were stained with 4′,6-diamidino-2-pheny-lindole (DAPI, Solarbio Science and Technology Co. Ltd.). CLSM was then used for the fluorescence observation.

### Singlet oxygen detection

Ethanolic solution of 1,3-Diphenylisobenzofuran (DPBF) (0.6 mg mL^−1^) was mixed with Ti_3_C_2_ and 3D CNT/MXene (equivalent Ti_3_C_2_ at 0.5 mg mL^−1^), respectively. UV-Vis spectra were recorded after irradiation times (650 nm, 0.2 W cm^−2^ and 808 nm, 1.5 W cm^−2^).

### Intracellular ROS detection

HeLa cells were seeded in confocal culture dishes at a density of 50,000 cells per well and incubated at 37°C under 5% CO_2_ for 24 h. Next, HeLa cells were incubated with 500 μL 3D CNT/MXene (0.5 mg mL^−1^) for 4 h. After incubation, cells were harvested and washed by PBS. The cells were then incubated with 2′,7′-dichlorofluorescein diacetate (DCFH-DA, 50 μM) for 20 min, followed by irradiation with 650 nm (0.2 W cm^−2^) and 808 nm (1.5 W cm^−2^) NIR laser for 10 min before imaging. DAPI was used to stain cell nuclei and CLSM was performed.

### Cytotoxicity test

The cytotoxicity of 3D CNT/MXene, 3D CNT/MXene-DOX, and DOX was determined by Cell Counting Kit-8 (CCK-8). Nine different groups HeLa cells were prepared corresponding to different experimental conditions [(I) control group; (II) control + 650 nm + 808 nm group; (III) free DOX group; (IV) 3D CNT/MXene group; (V) 3D CNT/MXene + 650 nm group; (VI) 3D CNT/MXene + 808 nm group; (VII) 3D CNT/MXene-DOX + 650 nm group; (VIII) 3D CNT/MXene-DOX + 808 nm group; (IX) 3D CNT/MXene-DOX + 650 nm + 808 nm group]. For each group, 5,000 HeLa cells were seeded in a 96-well plate. After 24 h of incubation, a fresh medium was added, and the cultures continued for another 24 h. The 3D CNT/MXene, 3D CNT/MXene-DOX, or control group were exposed to 650 and 808 nm wavelengths, as specified earlier. After that, the medium was replaced with CCK-8 solution, and the cells were incubated for 3 h. The absorbance of the supernatant was assessed using a DNM-9602 plate reader.

## Results and discussions

**FIGURE 1:**
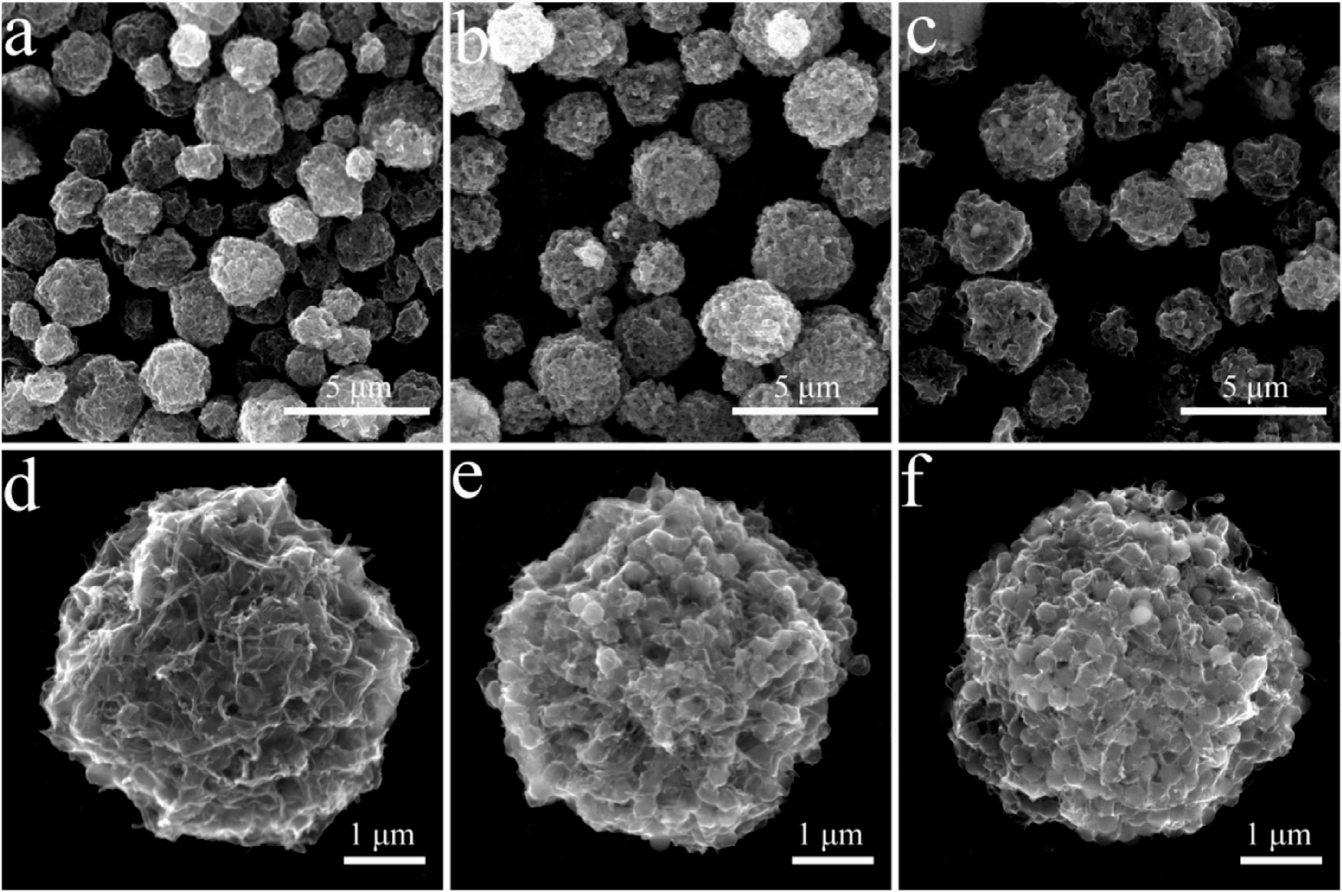
SEM images of 3D CNT/MXene@PMMA prepared with different ratios of CNT, MXene, and PMMA **(A,D)** 3D CNT/MXene@PMMA-8; **(B,E)** 3D CNT/MXene@PMMA-12; **(C,F)** 3D CNT/MXene@PMMA-16.

In this study, PMMA was used as the template and CNT/MXene as raw material to create 3D CNT/MXene@PMMA architecture *via* a spray-drying technique. The PMMA was then removed by calcination to afford 3D CNT/MXene. During the spray-drying process, the rapid evaporation of solvent from aerosol turned droplets into microspheres while maintaining the same sizes and regular shapes. Scanning electron microscopy (SEM) micrographs of 3D CNT/MXene@PMMA-8, PMMA-12, and PMMA-16 microspheres are shown in Figure 1. It is evident that PMMA microspheres are wrapped with MXene nanosheets, and CNTs are interspersed within them. The 3D CNT/MXene@PMMA was uniformly distributed with an average size of approximately 3–5 μm. With increasing PMMA content, the microspheres become more compact. However, the, microsphere with the CNT/MXene/PMMA mass ratio of 1:1:16 seemed slightly ruptured at some parts since the PMMA microsphere could not be wrapped entirely by the 3D CNT/MXene architecture due to its increased PMMA content.

**FIGURE 2:**
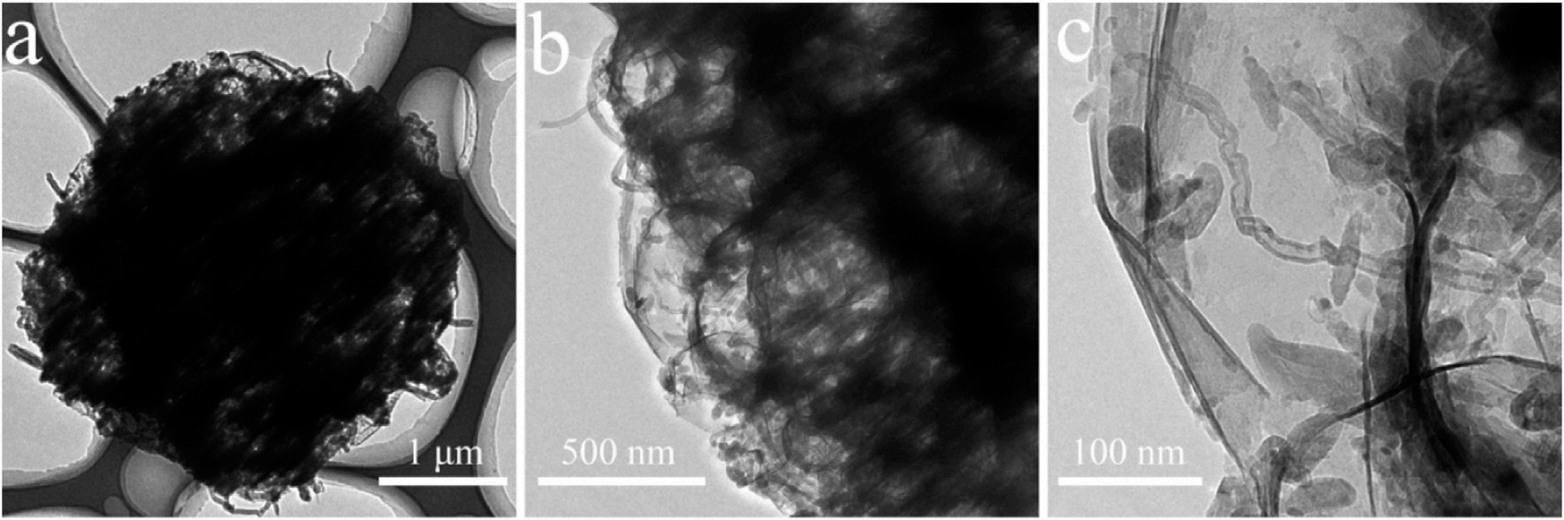
TEM images **(A–C)** of 3D CNT/MXene microspheres.

The morphology and microstructural properties of 3D CNT/MXene architecture were analyzed through TEM (Figure 2). The interior of the 3D CNT/MXene microspheres is hollow, and this cavity was retained after PMMA removal. Moreover, the 3D CNT/MXene structure did not changed from the 3D CNT/MXene@PMMA structure, and the CNTs were observed to be uniformly interspersed within MXene nanosheets. The tubular CNT structures and hollow MXene structure provide multiple possibilities for the slow-release of the loaded drug.

**FIGURE 3:**
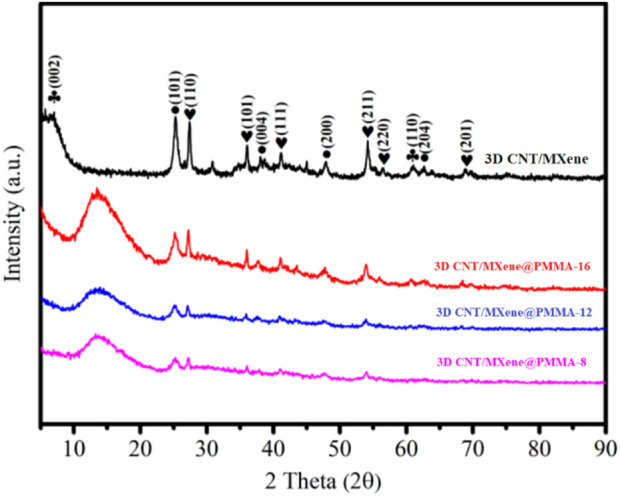
XRD patterns of the 3D CNT/MXene@PMMA-8, 3D CNT/MXene@PMMA-12, 3D CNT/MXene@PMMA-16, and 3D CNT/MXene composite.

The 3D CNT/MXene@PMMA-8, 3D CNT/MXene@PMMA-12, 3D CNT/MXene@PMMA-16, and 3D CNT/MXene were characterized using XRD (Figure 3). The 3D CNT/MXene@PMMA synthesized through this route displays typical CNT and PMMA peaks, indicating that PMMA microspheres are wrapped with MXene nanosheets. The 3D CNT/MXene exhibits similar characteristic diffraction peaks at 25.4°, 37.9°, 48.0°, and 64.1°, except for the (002) and (110) plane peaks of MXene, showing good agreement with standard anatase TiO_2_ (76–1,168, JCPDS). Additionally, there were six peaks of (110), (101), (111), (211), (220), and (201) planes corresponding to rutile phase TiO_2_ (76–1939, JCPDS). The XRD pattern shows distinctive TiO_2_ peaks, while the hump suggests that MXene is partially oxidized to form TiO_2_ during the spray-drying process. At the same time, the intensity of the diffraction peaks increases progressively as the content of PMMA increases. The PMMA peaks in 3D CNT/MXene, compared to 3D CNT/MXene@PMMA, are almost disappeared, suggesting the successful removal of the template of PMMA nanospheres by heat treatment. However, the presence of TiO_2_ peaks implies that TiO_2_ still exists on the surface of 3D CNT/MXene microspheres.

**FIGURE 4:**
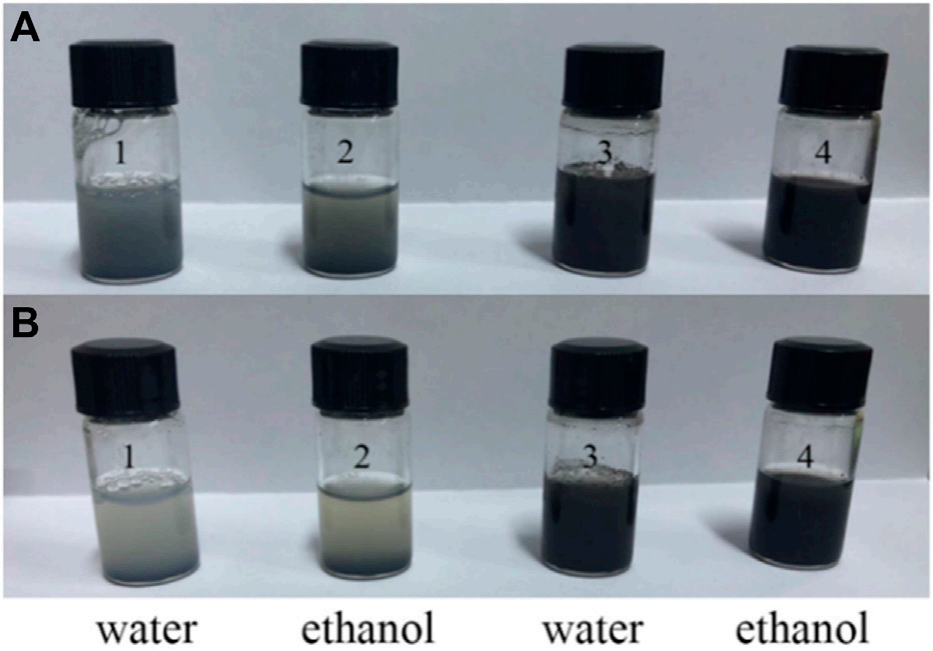
Dispersibility test of MXene and 3D CNT/MXene: After shaking **(A)**, and after resting for 2 h **(B)**; Samples 1 and 2 are MXene, Samples 3 and 4 are 3D CNT/MXene.

To investigate the anti-aggregation property of the 3D CNT/MXene, the dispersibility of MXene and 3D CNT/MXene in water and ethanol were tested. 3D CNT/MXene can be easily dispersed into an aqueous or ethanol solution through manual shaking and remains stable for more than 2 h, as shown in Figure 4. Conversely, MXene nanosheets precipitate rapidly in both solvents since the 2D materials nanosheets tend to aggregate through hydrogen bonds and van der Waals forces. Compared with 2D, the spherical 3D structure causes a reduction in the contact area of the 3D CNT/MXene, leading to the decreased adhesion forces. Therefore, the 3D CNT/MXene particles do not have close contact. Moreover, the van der Waal attraction is weak in the 3D structure, which helps the 3D CNT/MXene particles stabilize and disperse (FANG Y Z, et al., 2020).

**FIGURE 5:**
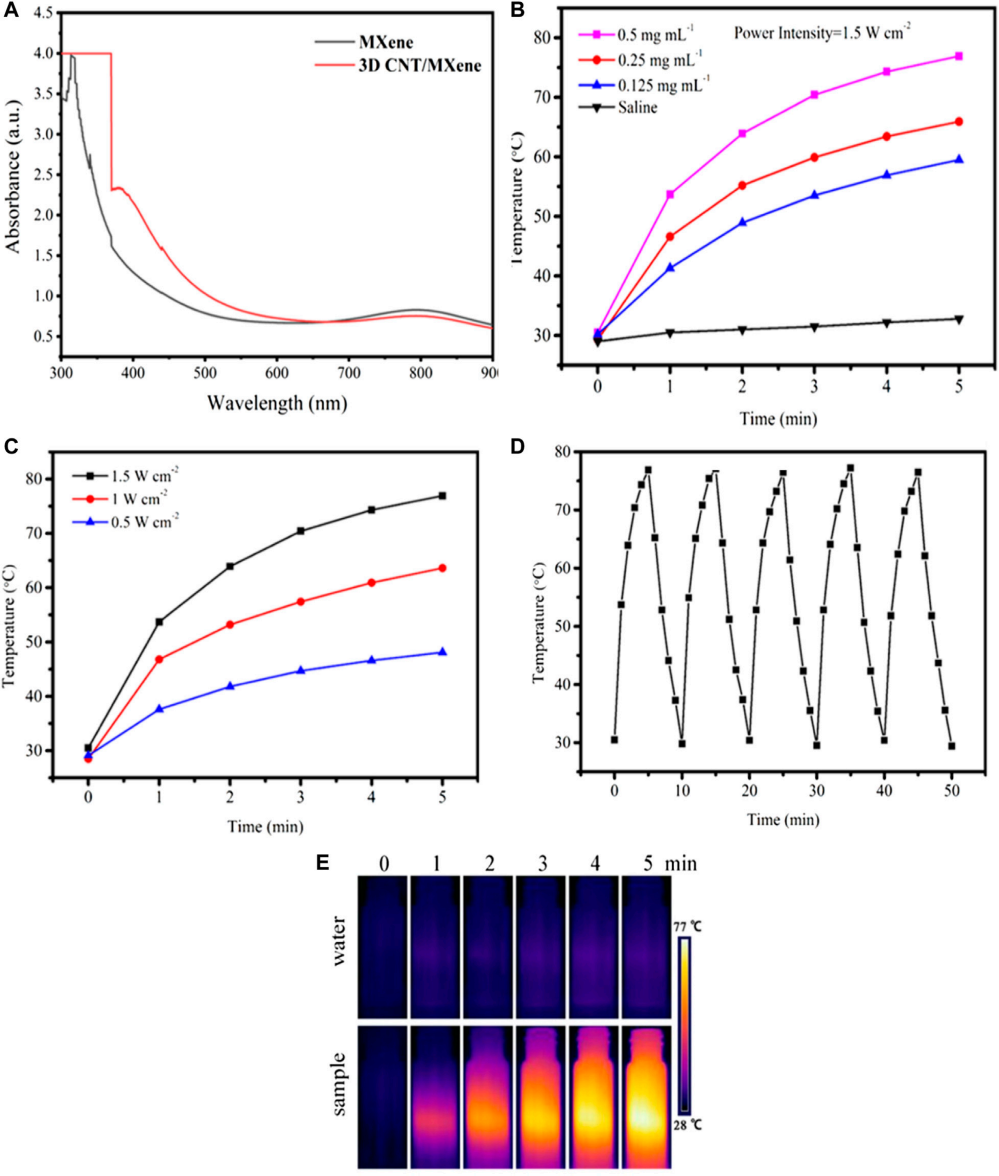
**(A)** UV-vis absorption spectra of MXene and 3D CNT/MXene, respectively. **(B)** Photothermal profile of 3D CNT/MXene solution at different concentrations (808 nm, 1.5 W cm^−2^). **(C)** Photothermal profile of 3D CNT/MXene solution (500 μg mL^−1^) under different laser power densities. **(D)** Photostability test of 3D CNT/MXene solution under 808 nm irradiation (1.5 W cm^−2^). **(E)** The infrared thermal images of 3D CNT/MXene at different time points under NIR laser irradiation.

It has been reported that the MXene nanosheets and CNT have excellent photothermal properties. An 808 nm NIR laser was chosen to investigate the photothermal properties of 3D CNT/MXene in this study since it can penetrate deeper and cause minor damage to the surrounding tissue. The UV-Vis spectrum of 3D CNT/MXene shows a unique absorption peak at a wavelength of about 808 nm (Figure 5A), making it a candidate for PTT photothermal agents. As displayed in Figure 5B, a gradual rise in temperature was observed within 5 min under 808 nm laser irradiation for all the solutions containing different concentrations of 3D CNT/MXene. The temperature reached 76.9°C for the 0.5 mg mL^−1^ dispersion liquid of 3D CNT/MXene. In contrast, the temperature of the deionized water was barely elevated under the same experimental conditions. The progressive temperature elevation of the 3D CNT/MXene dispersions was also observed with the increasing laser intensity (Figure 5C). Moreover, the 3D CNT/MXene dispersions have good photothermal cycling stability (Figure 5D). After irradiation for 30 min with the 808 nm laser, no significant photobleaching of 3D CNT/MXene dispersions was noticed. The photothermal effect of 3D CNT/MXene was also studied by *in vitro* temperature monitoring using an infrared thermal imaging camera. As shown in Figure 5E. The 3D CNT/MXene dispersion (500 μg mL^−1^) exhibited an increase in temperature from room temperature to 76.9°C after 5 min of NIR laser irradiation. The temperature change of 3D CNT/MXene dispersion is much more significant than the saline solution. According to the calculation formula of the photothermal conversion efficiency, the photothermal conversion efficiency of 3D CNT/MXene is about 82.9%. These results demonstrate that the 3D CNT/MXene possesses excellent photothermal properties and photothermal cycling stability.

**FIGURE 6:**
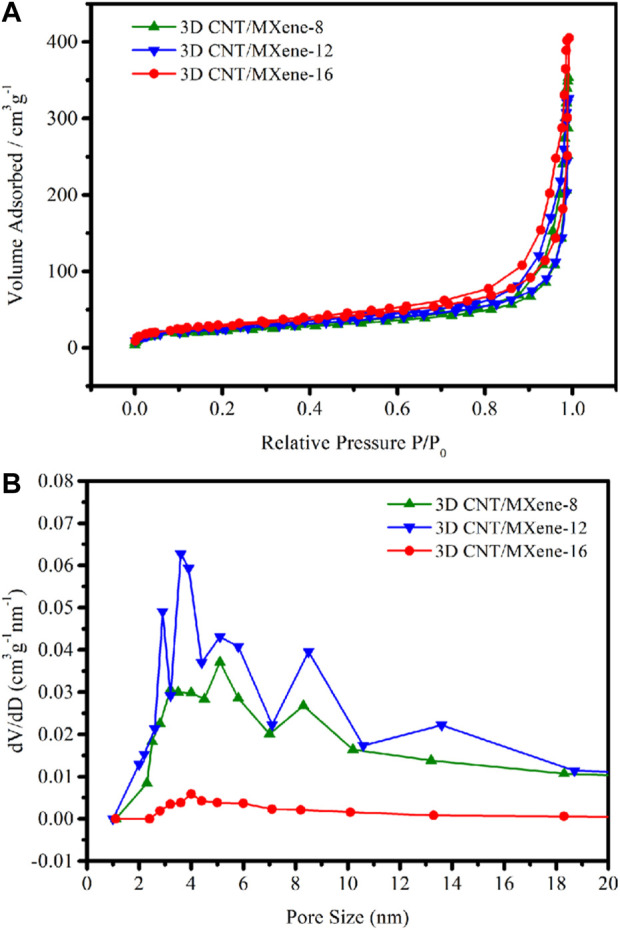
**(A)** N_2_ adsorption/desorption isotherms of 3D CNT/MXene-8, 3D CNT/MXene-12, and 3D CNT/MXene-16; **(B)** Pore diameter distribution of 3D CNT/MXene-8, 3D CNT/MXene-12, and 3D CNT/MXene-16.

The surface areas and the pore dimensions of 3D CNT/MXene-8, 3D CNT/MXene-12, and 3D CNT/MXene-16 were measured by using the BET and BJH methods, respectively (Figure 6). According to N_2_ physisorption data, all materials showed type-IV isotherms, demonstrating the mesoporous characteristic of 3D CNT/MXenes. The specific surface area of 3D CNT/MXene-8 (89.66 m^2^ g^−1^), 3D CNT/MXene-12 (98.61 m^2^ g^−1^), and 3D CNT/MXene-16 (108.08 m^2^ g^−1^) are found to be considerably higher than that of 2D MXene (44.5 m^2^ g^−1^), indicating that the obtained microspheres possessed a larger surface area, which was conducive for loading the anticancer drug (O Mashtalir, et al., 2014). 3D CNT/MXene microspheres of different mass ratios display peaks (i.e., bumps) in their pore size distribution curves (Figure 6B), corresponding to pore diameters ranging 2–10 nm. The DOX molecules can be readily loaded into the outer mesopore network of 3D CNT/MXene microspheres because of the large surface area of the mesoporous structures.

**FIGURE 7:**
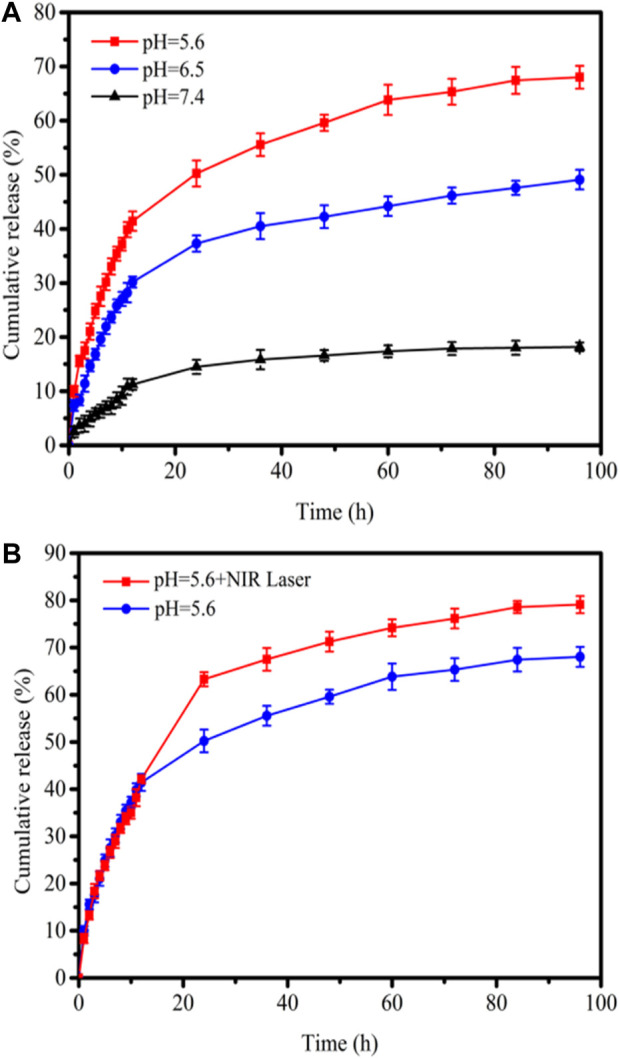
**(A)** DOX drug release profiles at pH 7.4, 6.5, and 5.6; **(B)** Drug release profiles with/without 808 nm laser irradiation at pH 5.6.

To demonstrate the acidic pH-responsive release of DOX from 3D CNT/MXene, the release kinetics of 3D CNT/MXene in PBS of pH 5.6, pH 6.5, and pH 7.4 were studied. The drug loading efficiencies were evaluated by measuring the UV-Vis absorbance of the supernatants. The 3D CNT/MXene has a high DOX loading capacity (85.6%) due to the high specific surface area, tubular and porous structures. As the tumor microenvironment is known to be acidic (pH 5.7–7.8), the synergistically enhanced photothermal effect in the acidic microenvironment could be used to treat cancer (E Veronica, et al., 2013). The DOX release at pH 7.4, 6.5, and 5.6 at different time points is illustrated in Figure 7A. When the pH changed from 7.4 to 5.6, the rate of DOX release increased markedly. The DOX release rate was much faster at pH 5.6, with 70.0% DOX release after 96 h, while only 15% DOX was released at pH 7.4. Thus, the pH-dependent release might enhance the target selectivity towards tumor cells since the acidic microenvironment of tumors may trigger DOX release from the 3D CNT/MXene-DOX. The high release rate of DOX in acidic environment can be attributed to the large solubility of DOX in acid, which is conducive to drug release, and more drugs are released after drug loaded microspheres enter the target site. Figure 7B shows that the increase of DOX release rate under NIR laser irradiation, plausibly because the electrostatic interaction was disrupted by the laser-induced photothermal effect to stimulate DOX release. Thus, 3D CNT/MXene-DOX has active tumor targeting abilities and high efficiency in drug delivery and release.

**FIGURE 8:**
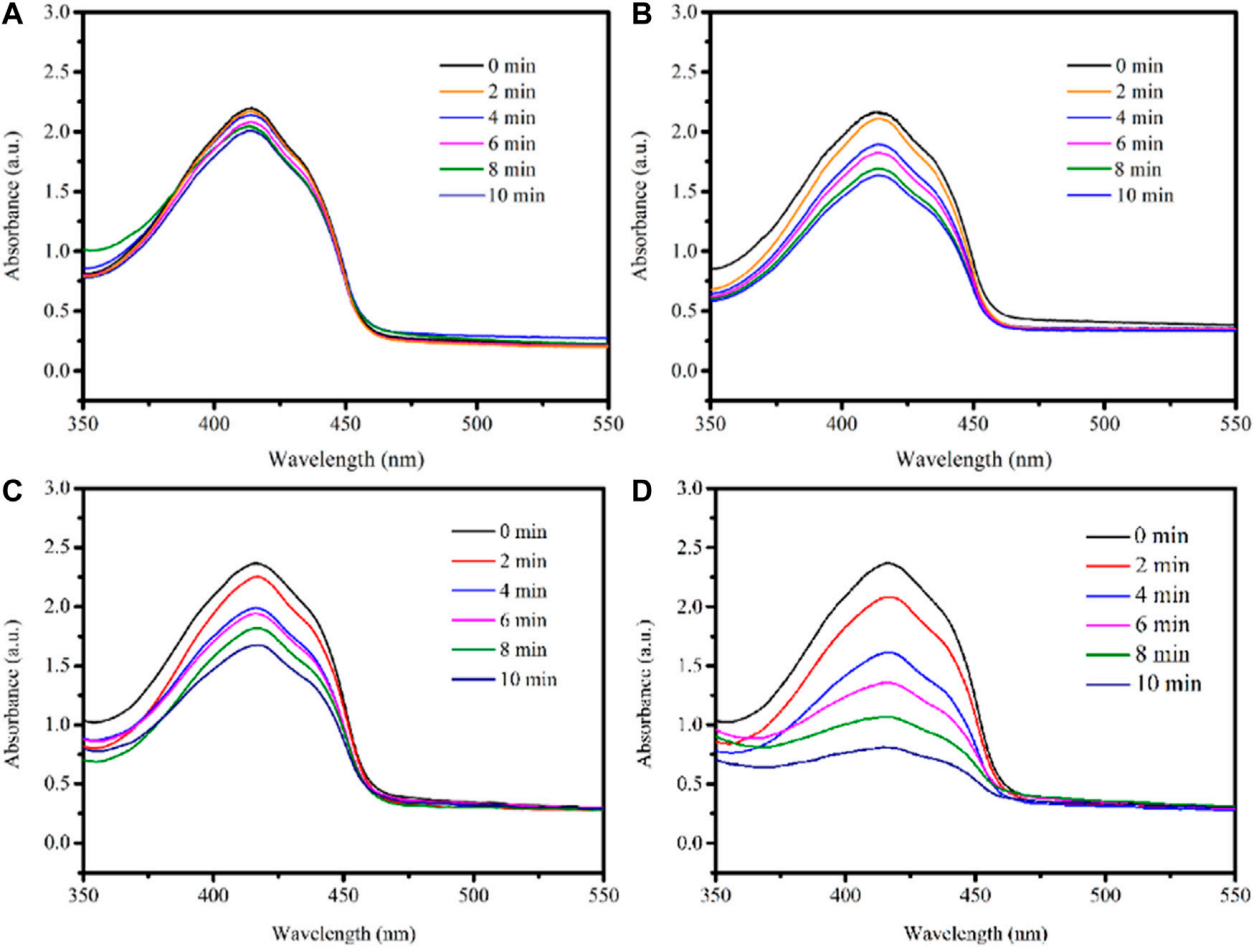
UV–Vis spectra of DPBF under laser irradiation: **(A)** DPBF+650 nm + 808 nm; **(B)** 2D MXene + DPBF+650 nm + 808 nm; **(C)** 3D CNT/MXene + DPBF+650 nm; **(D)** 3D CNT/MXene + DPBF+650 nm + 808 nm.

TiO_2_ has a broader bandgap, a low utilization rate for visible light, and absorbs only in the ultraviolet region, capable of damaging tissues. Using CNT as a sensitizer can extend the TiO_2_ absorption range to the visible light region. The generation of singlet oxygen (^1^O_2_), reactive oxygen species (ROS), was detected chemically using DPBF as a detector. As shown in Figure 8A, almost no reduction was observed in the absorbance of the DPBF solution at 650 and 808 nm irradiation. And there is a little reduction of the absorbance of DPBF solution in the presence of 2D MXene (Figure 8B). However, the absorbance of DPBF solution decays continuously upon 650 nm irradiation in the presence of 3D CNT/MXene (Figure 8C). The presence of TiO_2_ on the surface of 3D CNT/MXene and a larger specific surface area led to the decay in DPBF absorbance. The absorbance of DPBF decreased substantially in the presence of 3D CNT/MXene under 650 and 808 nm lasers irradiation (Figure 8D) demonstrating the significantly higher ability of 3D CNT/MXene to produce reactive oxygen species than the MXene nanosheets. Thus, as anticipated, the combination of TiO_2_ and CNT on the surface of 3D CNT/MXene enhanced its ROS generation ability, thereby making it a potential photodynamic therapy agent.

**FIGURE 9:**
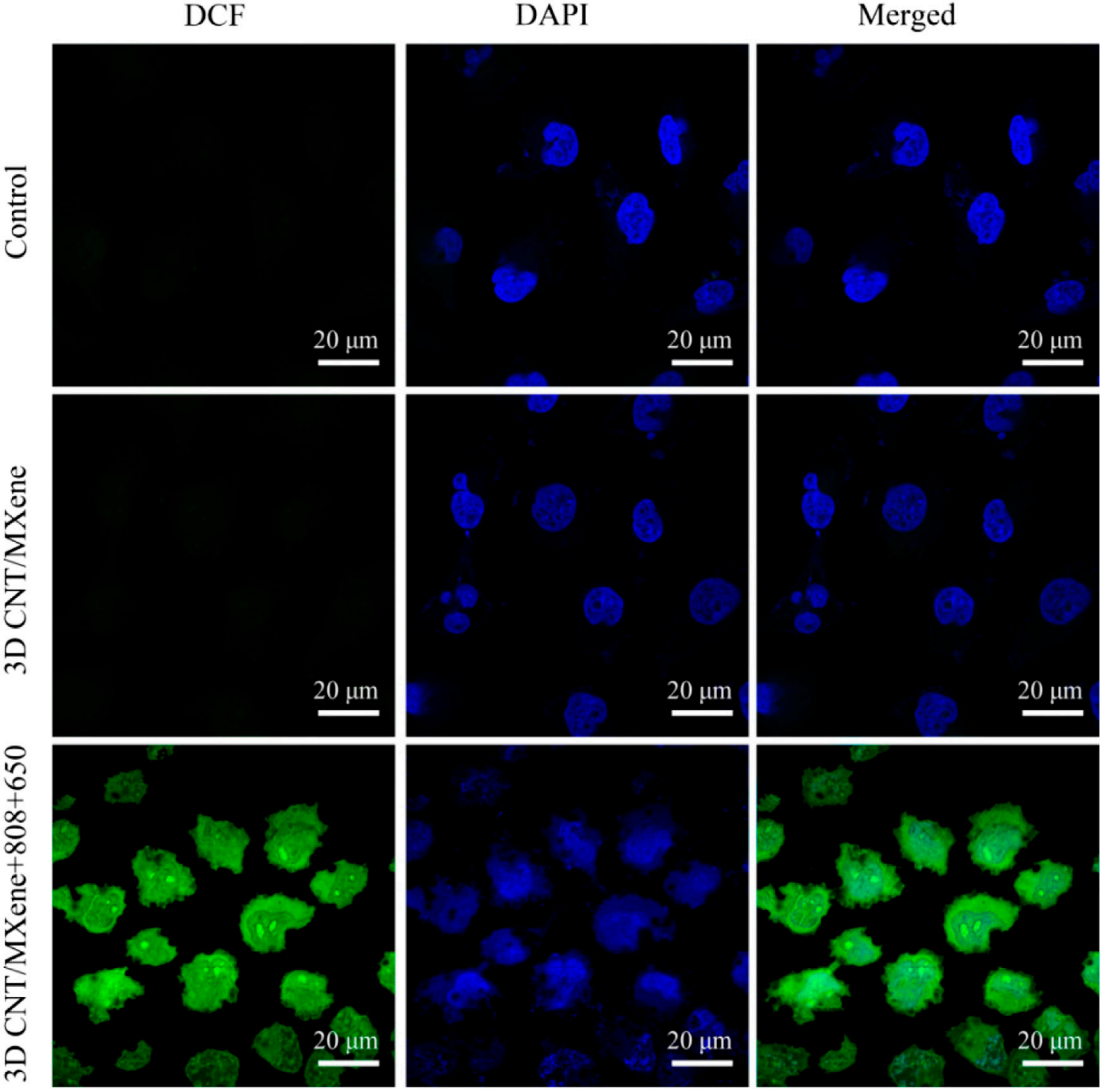
Fluorescent confocal microscopy images of HeLa cells that received different treatments.

To further assess the effect of 3D CNT/MXene on ROS production, ROS levels in HeLa cells were measured using DCFH-DA as a fluorescent probe. After co-incubating of 3D CNT/MXene microspheres with HeLa cells under different wavelength irradiation, an apparent green fluorescence is observed contrary to the control group (Figure 9), implying that 3D CNT/MXene produced significantly high levels of intracellular ROS.

**FIGURE 10:**
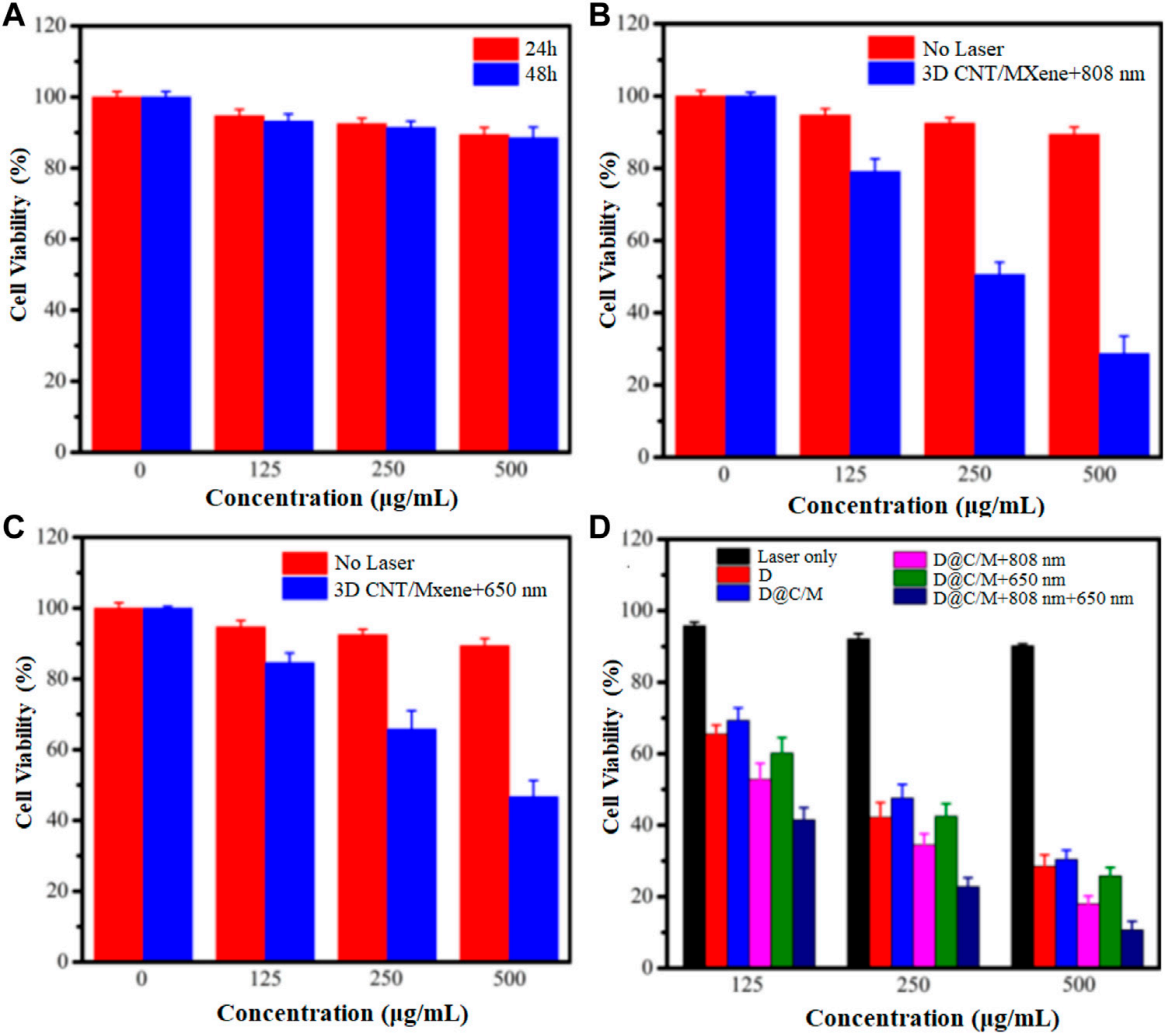
Viability of HeLa cells with varying concentration of 3D CNT/MXene in different environments **(A)** HeLa cells incubated with 3D CNT/MXene for 24 and 48 h; **(B)** HeLa cells incubated with 3D CNT/MXene without and with 808 nm laser irradiation; **(C)** HeLa cells incubated with 3D CNT/MXene at 650 nm laser irradiation; **(D)** HeLa cells incubated with 3D CNT/MXene with laser irradiation only, DOX only, D@C/M (D: DOX, C/M: 3D CNT/MXene), D@C/M+808 nm, D@C/M+650 nm, and D@C/M + 808 nm + 650 nm. The incubation time was 24 h.

The biocompatibilities of 3D CNT/MXene were investigated using a CCK-8 assay in human cervical cancer HeLa cells. As displayed in Figure 10A, HeLa cells were first incubated with different concentrations (0–500 μg mL^−1^) of 3D CNT/MXene for 24 and 48 h, and more than 88% cell viability was achieved under all culture conditions. The result suggests that 3D CNT/MXene microspheres possess no significant cell cytotoxicity and good biocompatibility. Photothermal cytotoxicity of various concentrations of 3D CNT/MXene microspheres towards cancer cells was accessed by their co-incubation with HeLa cells for 24 h under NIR laser irradiation. The HeLa cell inhibition rates for 3D CNT/MXene were 59.4% under NIR light stimulation (Figure 10B). The decreased cell viability rate is mainly due to the increased 3D CNT/MXene concentrations resulting in increased photothermal conversion effect and, hence, elevated temperature, which causes more cell death. The above results demonstrate the effectiveness of 3D CNT/MXene towards tumor destruction under laser irradiation. Hence, 3D CNT/MXene may be promising for photodynamic therapy in cancer treatment. Based on the excellent ^1^O_2_ generation ability of 3D CNT/MXene, the photodynamic effect of 3D CNT/MXene was studied by examining the phototoxicity *in vitro*. The inhibitory rate for 3D CNT/MXene was 44.7% for HeLa cells (Figure 10C), indicating that 3D CNT/MXene had a significant ^1^O_2_ generation ability. To further evaluate the synergic anti-tumor effect, the cell-killing effects of 3D CNT/MXene-DOX toward Hela cells under different wavelength irradiation were investigated. 3D CNT/MXene killed about 69.6% and 71.5% HeLa cells without NIR irradiation and after incubating with DOX, respectively, after 24 h (Figure 10D). Thus, DOX causes potential toxicity restricting its further application in tumor therapy. Additionally, the viability of HeLa cells upon co-incubation with 3D CNT/MXene (500 μg mL^−1^) reduced to 10.7% under 650 nm + 808 nm laser irradiation much lower than that of treated with 650 nm (25.8%) or 808 nm laser (18%) individually. These outcomes indicate that the synergistic anticancer effects of PTT/PDT/chemotherapy improve the therapeutic efficiency of 3D CNT/MXene against tumors.

## Conclusion

In conclusion, relatively simple, biocompatible 3D CNT/MXene-DOX nanoparticles as a synergistic therapy drug delivery platform were prepared. Upon 650 and 808 nm laser irradiation, the platform can be used in combined PDT/PTT therapy. 3D CNT/MXene-DOX microspheres exhibited excellent NIR-triggered PTT effect and perfect NIR photothermal stability. The ROS generation capacity of Ti_3_C_2_ and TiO_2_ revealed that using CNT improved conditions for TiO_2_ present on MXene surface by serving as carriers for TiO_2_ photosensitizers and exhibiting outstanding photodynamic therapeutic effects. DOX could be encapsulated into 3D CNT/MXene. 3D CNT/MXene-DOX displayed both NIR light and pH-responsive DOX release profiles *in vitro*, enhancing the therapeutic anticancer effect. Furthermore, the 3D CNT/MXene-DOX system facilitates chemo-drug delivery and provides a new guiding strategy for developing Ti_3_C_2_-based synergistic PTT/PDT/chemotherapy for tumor treatment. Therefore, 3D CNT/MXene with efficient PDT and PTT effects are a promising multifunctional cancer therapy platform.

## Data Availability

The original contributions presented in the study are included in the article/Supplementary Material, further inquiries can be directed to the corresponding authors.
